# The Impact of Online-Schooling during COVID-19 on Device-Measured 24-Hour Movement Behaviours among High School Students: A Compositional Data Analysis

**DOI:** 10.3390/children9050667

**Published:** 2022-05-05

**Authors:** Petra Starbek, Kaja Kastelic, Nejc Šarabon

**Affiliations:** 1 Alma Mater Europaea—European Center Maribor, 2000 Maribor, Slovenia; petra.starbek@gmail.com; 2 Andrej Marušič Institute, University of Primorska, 6000 Koper, Slovenia; kaja.kastelic@iam.upr.si; 3 InnoRenew CoE, 6310 Izola, Slovenia; 4 Faculty of Health Sciences, University of Primorska, 6310 Izola, Slovenia

**Keywords:** SARS-CoV-2, time-use epidemiology, sleep, sedentary behaviour, physical activity, youth, adolescents

## Abstract

The COVID-19 measures have unfavourably affected the movement behaviours of youth. The aim of this study was to explore the impact of online-schooling during COVID-19 on device-measured sleep (SL), sedentary behaviour (SB), light physical activity (LPA), and moderate–vigorous physical activity (MVPA) among high school students. A total of 51 students (26 female) from Slovenia wore an activity monitor activPAL4 during the weekdays of onsite-schooling and during the weekdays of online-schooling. Data on movement behaviours were analysed using compositional data analysis. During the onsite-schooling (and online-schooling), students spent on average 432 min/day (469 min/day) in SL, 731 (755) in SB, 253 (202) in LPA, and 25 (15) in MVPA. Repeated measures multivariate analysis of variance confirmed significantly more time was spent in SL (log ratio 0.29; 95% CI 0.20, 0.37)) and SB (log ratio 0.23; 95% CI 0.13, 0.32) and less time in MVPA (log ratio −0.44; 95% CI −0.66, −0.23) during the online-schooling weekdays. Students spent significantly more time in SB during leisure (log ratio 0.20; 95% CI 0.06, 0.35) as well as during lecture time (log ratio 0.45; 95% CI 0.32, 0.58). Our results indicate that online-schooling significantly contributed to the unfavourable changes in students’ movement behaviours during COVID-19. Future studies should focus on developing physical activity interventions suitable for the circumstances of the epidemic.

## 1. Introduction

In early 2020, the novel coronavirus (SARS-CoV-2) that emerged in Wuhan, China rapidly spread across all the continents, and it severely affected most countries. The World Health Organization (WHO) declared a COVID-19 pandemic on 11 March 2020 [1]. In order to reduce the spread of the novel virus, the countries adopted different measures, such as physical distancing, closure of non-essential services, and recommendations to stay at home, including working and schooling from home. Though the established COVID-19 measures helped to save many lives [2], they had some unfavourable consequences to public health as well. People changed their daily routines, including health-related behaviours. Particularly, studies reported that during the COVID-19 pandemic the overall physical activity (PA) decreased, and the time spent in sedentary behaviour (SB) and sleep (SL) increased [3,4].

Public health concerns have been raised particularly by the decreased levels of PA, since it could already have an adverse effect on physical and mental health in the short term [5,6,7]. There is also a potential risk that the behavioural changes adopted during the pandemic would become permanent, importantly affecting public health also in the long term. Specifically, adolescents may be most susceptible to the permanent adoption of pandemic-induced adverse changes in health-related behaviours since behavioural patterns and psychomotor abilities are under intense development during this phase of life.

In the pre-pandemic period, adolescents spent most of the time being physically active during physical education, organised sports, active transport to and from school, and during time spent in recreational facilities and parks [8,9,10]. With the closures of schools and recreational facilities and stay-at-home guidelines during the pandemic, adolescents were left with fewer opportunities to be physically active. Moreover, the above-mentioned restrictions potentially led to less structured daily schedules, such as those observed during the weekends and school breaks, when students are less physically active [11,12]. 

Currently, little is known about the impact of specific COVID-19 measures (e.g., school closures, organised sports cancellation, outdoor exercise/playing prohibition) on movement behaviours among adolescents. Most of the studies reported a decrease in overall PA [3,13,14], with greater changes observed in countries where COVID-19 measures included school closures and permission to leave home only for essential needs (e.g., China and Spain). However, a study conducted in Germany, where outdoor exercise/play was allowed during the COVID-19 measures but the schools were closed, reported an increase in total PA among adolescents [15]. The authors argued that observed changes may be related to the fact that adolescents had more free time due to the school closures. 

It is important to understand the impact of specific epidemic-related restrictions on the movement behaviours of adolescents. Such evidence could inform future movement behaviour interventions aiming to combat the impact of epidemic-related policy restrictions on the behaviours of adolescents or even influence decisions regarding future restrictions. Therefore, the aim of this study was to explore the impact of online-schooling during COVID-19 measures on levels of SL, SB, light PA (LPA), and moderate–vigorous PA (MVPA) among high school students. We hypothesised that students were less physically active and spent more time in SB and SL during COVID-19 measures that included online-schooling (attending classes from home) compared to traditional onsite-schooling conditions (attending classes at school).

## 2. Methods

### 2.1. Participants’ Recruitment

A convenience sample of students was recruited from five different high schools located in the central region of Slovenia. To be included in the study, participants had to be generally healthy, able to move without any physical limitations, and attend high school during COVID-19 measures. Those attending the final year of high school were not recruited for the study due to the untypical school schedule arising from preparations for final exams. At the time when the study was conducted (during the third COVID-19 wave in the country, when a 7-day moving average of daily new COVID-19 cases was approximately 1000 (50 new cases per 100,000, April–May 2021), Slovenian students participated in a hybrid model of schooling that was a part of COVID-19 measures in the country. Students attended classes at school (i.e., traditional onsite-schooling conditions) in one week (Monday to Friday) and then attended online classes from home (i.e., online-schooling conditions) in the following week (Monday to Friday), etc.

The study was conducted in accordance with the Declaration of Helsinki, and it was approved by the Republic of Slovenia National Medical Ethics committee (approval number: 0120-63172017/2). All participants and their guardians signed an informed consent before the beginning of the study. The required sample size to achieve the statistical power of 95% in repeated measures MANOVA (within factors design, *p* < 0.05, *r* = 0.5), if the true difference in the population is of at least a medium size (effect size *f* = 0.25) is 54 participants. To allow for a 10% attrition of the initial study sample, we recruited 60 participants. However, during the last week of our study, the COVID-19 measures that included a hybrid model of schooling were removed, leading to eight participants that could not complete the study protocol. Out of the remaining 52 participants, all but one successfully completed the study protocol, and they were included in the further analysis.

### 2.2. Measurements

Movement behaviours (i.e., MVPA, LPA, SB, and SL) were measured using the activity monitor activPAL4 micro (PAL Technologies Ltd., Glasgow, Scotland). ActivPAL4 is a light (9 g) and small (55 × 25 × 5 mm) inclinometer-based accelerometer that is worn on the anterior aspect of the right thigh. Using a proprietary software (PAL analysis, PAL Technologies Ltd., Glasgow, UK), it can identify periods of sitting, lying, standing, and stepping [16].

The onset (hh:mm) and the offset (hh:mm) of SL time and school time were measured using a time-use paper-based diary. For each calendar day, participants were required to complete the following information: (i) at what time they woke up, (ii) at what time their school classes started, (iii) at what time their school classes ended, (iv) at what time they fell asleep, and (v) the time period when they had a daytime nap, if applicable. Responses were provided as date (dd-mm-yyyy) and time (hh:mm). The participants were asked to complete the diary on a daily basis.

Socio-demographic data were provided by the participants using an online questionnaire that asked about their sex (*male/female*), age (*years*), body height (*cm*), body weight (*kg*), year of high school (*1st/2nd/3rd year*), smoking (*yes/no*), residence (*urban/rural*), and socio-economic status (*high or very high/middle/low or very low*). Body mass index (BMI) was calculated from self-reported body height and body weight, and the WHO’s cut-offs were used for the interpretation of students’ BMI (thinness: <−2 SD; normal: −2 SD to +1 SD; overweight: >+1 SD) [17].

### 2.3. Study Design

This study had a repeated measures design where participants’ 24-h movement behaviours were observed under two different conditions: attending classes at school and attending online classes (which were part of the established COVID-19 measures in Slovenia at the time the study was conducted). Before the study began, participants were invited to meet the researcher who presented the study protocol and answered questions raised by the participants. Afterwards, the researcher placed the activPAL4 (inside of the flexible waterproofed sleeve) on the anterior aspect of participants’ right thigh (midway between the anterior superior iliac spine and the knee) using a hypoallergenic adhesive dressing (Tegaderm). Participants were asked to wear the activPAL4 continuously for 14 consecutive days, while removing the device only in case of swimming or sauna (any device removal was requested to be reported within the diary). The activPAL4 default recording mode (10-bit resolution, 20 Hz sampling frequency, ±4 g range of acceleration) was chosen for the data collection. 

Participants were also introduced to the time-use diary, and they were requested to complete the diary daily. Afterwards, they completed an online socio-demographic questionnaire. For the case of any additional questions or difficulties that may arise during the measurement period, the researcher provided their telephone number. Participants also received two additional adhesive dressings along with the paperback instructions on how to replace the adhesive. After the measurement period, participants returned the devices and completed time-use diaries.

### 2.4. Data Management

The data from the activPAL4 were downloaded and processed using proprietary software (PAL connect and PAL analysis, version 8.11.5.64, PAL Technologies Ltd., Glasgow, UK). The CREA algorithm was used to process the data and to compute the outcome variables. A midnight-to-midnight approach was chosen to define a day, and a 24-h protocol that allowed for up to 4 h of non-wear time was adopted as a criterion for a valid day. The self-reported periods (hh:mm when participant fell asleep and hh:mm when they woke up) from the time-use diary were manually entered into the PAL analysis (in primary time-in-bed view) to differentiate between sitting/lying while awake and SL. The daily summary spreadsheet that contains summary data for each valid day (for each participant) of the recording was then exported as a .csv file. To be included in the analyses, the participant needed to provide at least 3 valid weekdays of activPAL4 data from days when they attended classes at schools, and at least 3 valid weekdays when they attended online classes [18].

SL time was calculated by summing activPAL4 output called “primary lying time” (that was set based on entered hours of self-reported SL time) and self-reported napping time. Sedentary time was calculated by summing activPAL4 output “sitting time” and “secondary lying time”, while deducting self-reported napping time. A step cadence method (≥100 steps per minute) was used to assess the time spent in moderate-to-vigorous physical activity (MVPA) [19], which was provided within the activPAL4 .csv export file (daily summary spreadsheet). Lastly, light physical activity (LPA) was calculated as a residual time to wear time (LPA = 24 h—SL—SB—MVPA—non-wear time). Weekend days were not included in the analysis.

To analyse the periods when the participant attended school classes (i.e., lecture time), the individual activPAL4 events files were exported from the PAL analysis software and imported into the PAL event analysis tool [20]. This tool is a Microsoft Excel spreadsheet that can process the file of individual events and produce a summary of activity data of custom-defined periods. To calculate the activity data for daily periods when the participant did not attend classes (i.e., leisure time), the activity data from periods with attended school classes were deducted from the daily summaries. If non-wear time existed, the data on lecture/leisure movement behaviours were proportionally rescaled to fit a specific time period [21].

### 2.5. Statistical Analysis

The data were analysed in R version 4.0.5 [22] and R Studio 1.4.1106 [23], using the packages “tidyverse”, “compositions”, “rstatix”, “Hmisc”, “dplyr”, “ggplot2”, “gridExtra”, and “grid”. Socio-demographic data were presented as absolute and relative frequencies (%).

Compositional data analysis (CoDa) was used to present and to analyse movement behaviour data [24,25,26,27]. CoDa is a branch of statistics developed specifically for data that are compositional by nature (i.e., data that are mutually exclusive and exhaustive parts/proportions of a finite total). For example: SL, SB, LPA, and MVPA are parts of a 24-h day. They are perfectly collinear and intrinsically co-dependent, which means that change in time spent in one behaviour will necessarily result in a corresponding change in other behaviour(s); and CoDa successfully deals with specific mathematical properties of such types of constrained data. In CoDa, a measure of the central tendency that best describes the data is the compositional mean (for equations see [28]). When aiming to apply the inferential statistics, the first procedure in CoDa is usually to transform data using the isometric log-ratio (ilr) transformation that returns data that are not constrained, and as such it can be processed using standard statistical techniques [29].

In our study, two 24-h time-use compositions were constructed: the first one consisted of 4-parts of relative amounts of time spent in SL, SB, LPA, and MVPA, and the second one consisted of 6-parts of relative amounts of time spent in SL, leisure time SB, LPA and MVPA, and lecture time SB and PA (we merged lecture time LPA and MVPA into a PA category, since a substantial proportion of students (4% while onsite-schooling and 39% while online-schooling) did not accumulate any MVPA minutes during lecture time and we considered it as true zeroes). We calculated compositional means for both 24-h time-use compositions. To visualise the difference between the conditions tested, we constructed the geometric mean bar plots on log-ratios (detailed description of a procedure can be found elsewhere [24]) between compositional means, while online-schooling and onsite-schooling conditions for the 24-h movement behaviours (4-part composition) and for 24-h domain-specific movement behaviours (6-part composition).

The ilr data transformation was used on both 24-h time-use compositions. For the first 4-part composition, the ilr coordinates were calculated as [25]:

(1) $$\mathrm{ilr}1=\sqrt{\frac{3}{4}}\;\ln\;(\frac{\left(\mathrm{SL}\right)}{\sqrt[{3}]{\left(\mathrm{SB}\times \mathrm{LPA}\times \mathrm{MVPA}\right)}})$$

(2) $$\mathrm{ilr}2=\sqrt{\frac{2}{3}}\;\ln\;(\frac{\left(\mathrm{SB}\right)}{\sqrt[{2}]{\left(\mathrm{LPA}\times \mathrm{MVPA}\right)}})$$

(3) $$\mathrm{ilr}3=\sqrt{\frac{1}{2}}\;\ln\;(\frac{\left(\mathrm{LPA}\right)}{\left(\mathrm{MVPA}\right)})$$

where ilr1 presents the ratio of SL time to time spent in the waking behaviours (i.e., SB, LPA, and MVPA); ilr2 presents the ratio of time spent in SB to time spent in physical activity (i.e., LPA and MVPA); and ilr3 presents the ratio of time spent in LPA to time spent in MVPA. Repeated measures multivariate analysis of variance (RM MANOVA) was applied to the ilr coordinates to test the difference in 24-h movement behaviours between onsite-schooling and online-schooling conditions [24,30]. Then, the additional three first ilr coordinates were calculated, where SB, LPA, and MVPA were placed (alone) in the nominator and the remaining three behaviours in the denominator. Post hoc tests (using the paired sample *t*-test) were performed on all four first ilr coordinates to explore the difference in each movement behaviour (relative to the remaining behaviours) between the conditions tested. Cohen’s *d* was used as a measure of effect size. Cohen’s *d* of 0.2, 0.5, and 0.8 were considered small, medium, and large, respectively [31]. A level of significance was set at α < 0.05. The RM MANOVA was also applied to the 6-part domain-specific movement behaviours. The post hoc tests were performed for the two sub-compositions (including leisure time and lecture time movement behaviours) to explore the relative differences in movement behaviours during leisure time and lecture time between the two conditions.

## 3. Results

Fifty-one (26 female) students successfully completed the study protocol and were included in the further analysis. The participants’ mean age was 16.4 ± 1.1 years (ranged from 15 to 19 years of age). Most participants had normal zBMI, were non-smokers, lived in an urban area, and had a middle socio-economic status. Detailed participant characteristics can be found in Table 1. None of the participants were infected with COVID-19 or under self-isolation due to high-risk contact while participating in the study. Participants provided 220 valid days of activPAL4 data for online-schooling condition, and 196 valid days of activPAL4 data for onsite-schooling condition, with minimal non-wear time (in total, only 2.7 h within 416 valid days).

**Figure 1:**
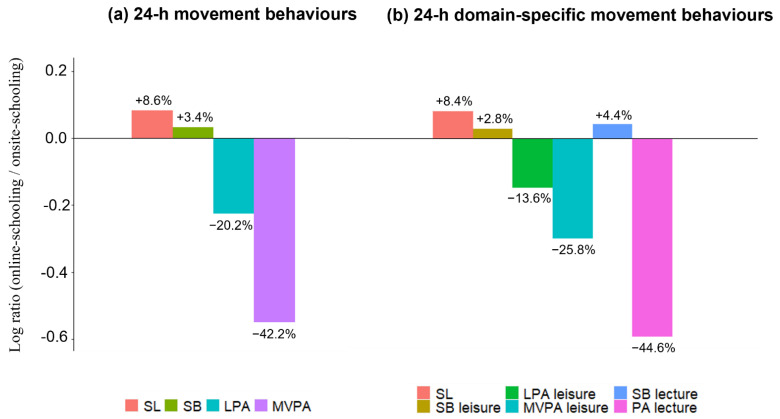
Geometric mean bar plot on log-ratios between compositional means while online-schooling and onsite-schooling conditions for (**a**,**b**). For example: geometric mean bar plot (**a**) indicates that on average students spent more time in SL (+8.6%) and SB (+3.4%), and less time in LPA (−20.2%) and MVPA (−42.2%) on days when they attended lectures from home (online) compared to when they attended lectures from school (% were calculated as: exp of log ratio × 100–100). Abbreviations: SL, sleep; SB, sedentary behaviour; LPA, light physical activity; MVPA, moderate-to-vigorous physical activity; PA, physical activity.

Compositional means of a 4-part composition of daily time spent in SL, SB, LPA, and MVPA during onsite-schooling (and online-schooling) conditions were 431.9 min/day (468.9 min/day), 730.5 min/day (755.0 min/day), 252.5 min/day (201.5 min/day), and 25.2 min/day (14.5 min/day), respectively (Table 2). RM MANOVA revealed a significant difference between onsite-schooling and online-schooling condition in the 24-h movement behaviours (Wilks = 0.395, F_3,48_ = 24.461, *p* < 0.001). Post hoc tests (using the paired sample *t*-test) on the first ilr coordinates revealed a significant difference for time spent in SL, SB, and MVPA (all relative to the remaining behaviours) (*p* < 0.001 for all), with a large effect size for SL (*d* = 0.956) and moderate effect sizes for SB (*d* = 0.669), and MVPA (*d* = −0.575). According to log-ratios between the compositional means of the two conditions (Figure 1), students’ time spent in SL and SB increased by 8.6% and 3.4%, respectively, while time spent in LPA and MVPA decreased by 20.2% and 42.2%, respectively, when online-schooling compared to when onsite-schooling. However, the decrease in LPA relative to the remaining three behaviours was not statistically significant (*p* = 0.086). 

Additionally, we explored the differences for domain-specific movement behaviours by partitioning daily movement behaviours into a 6-part composition of SL, leisure time SB, LPA, and MVPA, and lecture time SB and PA. Compositional means are presented in Table 2. RM MANOVA revealed a significant difference between onsite-schooling and online-schooling condition in domain-specific movement behaviours (Wilks = 0.339, F_5,46_ = 17.924, *p* < 0.001). Post hoc tests (using the paired sample *t*-test) on the first ilr coordinates for a 3-part sub-composition of leisure time movement behaviours revealed a significant difference only for time spent in SB relative to the remaining leisure time behaviours (*p* < 0.007), with a small effect size (*d* = 0.396). A post hoc test (using the paired sample *t*-test) on the first ilr coordinates for a 2-part sub-composition of lecture time movement behaviours revealed a significant difference for time spent in SB and PA (both relative to the remaining lecture time behaviour) (*p* < 0.001), with a large effect size (*d* = 0. 984). 

## 4. Discussion

The key findings from this study are that during the COVID-19 measures, high school students got on average less than 8 h/day of SL, spent more than 12 h/day in SB, and engaged in less than 0.5 h/day in MVPA. Moreover, high school students slept longer and spent significantly more time in SB and less time in PA during the weekdays when they attended online classes, compared with the weekdays when they attended classes at school. A detailed analysis revealed that during the online-schooling conditions, students spent significantly more time in SB during leisure, as well as during lecture time. 

### 4.1. Comparison with Previous Studies

To the best of our knowledge, this was the first study that explored the impact of online-schooling on students’ movement behaviours during the COVID-19 measures. We found that students slept longer (+8.6%), spent more time in SB (+3.4%), and less time in LPA (−20.2%) and MVPA (−42.2%) during the COVID-19 measures that included online-schooling. The directions of observed changes are in accordance with studies that explored the overall effect of COVID-19 measures on youth’s movement behaviours, showing that SL time and SB increased while PA decreased [3]. Specifically, studies that compared pre- and during-pandemic levels of movement behaviours of the youth reported an increase in SL between 7.0% and 21.4% [32,33,34,35], an increase in SB between 6% and 52% [35,36,37,38,39], and a decrease in LPA between 32.5% and 30.0% [36,37,38,40,41] and in MVPA between 3.9% and 56.6% [42,43,44,45].

The magnitude of the changes in movement behaviours of the youth has likely been influenced by the nature and the severity of the established COVID-19 measures. For example, Lopez-Bueno et al. [44] reported that Spanish adolescents slept significantly longer during the period of strict confinement (9.3 h/day) compared to the period of more relaxed confinement (9.0 h/day). Moreover, greater changes in PA and in SB during the COVID-19 measures were reported from countries where more strict measures, such as school closure and stay-at-home guidelines, were established [3,13,14]. At the time our study was conducted, the COVID-19 measures in the country included wearing masks and physical distancing, recommendations to work from home and stay at home, prohibition to leave the region of residence, cancellation of sports events, closure of playgrounds, prohibition of gatherings, and closures of bars, restaurants, and other unessential services. Schools adopted a hybrid model of schooling that included weekly shifting of at school classes and online classes. Online-schooling is one of the strictest epidemic-related measures, and our study revealed that it has a significant effect on students’ 24-h movement behaviours.

It is very likely that other established measures also influenced students’ movement behaviours. However, since we did not evaluate movement behaviours before the COVID-19 measures were established, we can only speculate on the relative contribution of online-schooling to overall changes in students’ movement behaviours. For example, the studies conducted before the COVID-19 pandemic (that also used activPAL, as in our study) reported that their Scotland peers spent on average 8.9 h/weekday in SB [46], and the Australian peers 9.6 h/weekday [18]. In our study, students spent on average 12.2 h/weekday in SB during onsite-schooling and 12.6 h/weekday during online-schooling. Based on the comparison of our results with previous studies, we could speculate an increase in SB of around 3.0 h/weekday during the COVID-19 measures that excluded online-schooling and of approximately 3.4 h/weekday during the COVID-19 measures that included online-schooling. A similar estimate of changes in SB from pre- to during-pandemic period was also reported for university students [35]. It may be that a combination of other COVID-19 measures (e.g., physical distancing and prohibited gatherings, cancelled sports events, closed playgrounds, and stay-at-home encouragements) had a greater relative contribution to the overall change in SB than online-schooling. Nevertheless, our results show that high school students were highly sedentary during the COVID-19 measures.

Our results also reveal that high school students were more sedentary during leisure as well as during lecture time while online-schooling. Those findings are in accordance with a study conducted on the adult population, showing that employees increased their work-related sitting time and recreational screen time during the COVID-19 confinement when working from home was more prevalent [47]. Attending online classes from home may provide fewer opportunities/motives for standing and other PA. For example, when in the school environment, students are often upstanding during class breaks—to interact with other students and while moving to another classroom, during active lessons, and while having a physical education class. Such opportunities were likely compromised while attending online classes. Moreover, students did not have to commute to school while online-schooling, and for those who normally engage in active commuting, which is an important contributor to their leisure time PA [48], this opportunity to be active was lacking. It was reported previously that while online-schooling, students slept longer in the morning and fell asleep 120 min earlier [35], indicating that they substituted morning commuting time with SL time. This may partly explain our findings that students slept longer while online-schooling. Last but not least, a lack of afternoon commuting while online-schooling likely increased students’ leisure time, and previous studies suggested that students preferred spending extra leisure time during COVID-19 measures engaging in sedentary screen time activities [15,44]. A lack of afternoon commuting has probably also limited spontaneous social interactions/activities. This may partly explain our findings that students spent more leisure time in SB while online-schooling.

### 4.2. Practical Implications

Our findings indicate that online-schooling contributed substantially to the less healthy 24-h movement behaviours among students during the COVID-19 measures. Interventions that aim to decrease the levels of SB and, particularly, increase MVPA are warranted in future lockdowns, especially if the restrictions also include online-schooling. Interventions are needed for leisure, as well as for lecture time. It might be worth raising the awareness of parents/guardians, who can influence adolescent’s movement behaviours by setting rules (e.g., screen time limits, household obligations, and sleep hygiene) and by acting as a role model for a healthy time-use. Teachers may also play an important role in encouraging students to sit less and move more by integrating regular active breaks during the online classes and by assigning students schoolwork and homework that require them to be more physically active. Countries and policies should support such approaches by providing a safe (indoor and outdoor) space where the youth could be active during the potential future lockdowns.

### 4.3. Strengths and Limitations

The major strength of our study was that we used the activity monitor activPAL4 to estimate the levels of movement behaviours during the COVID-19 measures, and that we used compositional data analysis. Moreover, the wear time compliance in our sample was very high, which also contributed to the higher accuracy of the estimates. However, some limitations must be acknowledged. Firstly, this study included a convenience sample that was not fully representative (e.g., students in our sample attended general secondary education programs only), indicating the findings may not be generalizable to other sub-populations (e.g., students attending vocational secondary education programs). Secondly, our study has an observational longitudinal design, which is not the most rigorous approach for identifying causal effects. However, such designs are common in behavioural/social science (where randomisation of individuals is often impossible or unethical) to evaluate the causality, since they present the evidence on temporality that is an important criteria for establishing causal relationships [49]. Thirdly, the findings should not be generalised to online-schooling conditions other than in the context of the pandemic. Fourthly, we relied on self-reported data on the onset and the offset of sleep, leisure, and lecture time that may be affected by recall bias. Lastly, given the relatively small sample size, we were not able to do the stratification analysis (e.g., analysis by sex, age, BMI).

## 5. Conclusions

High school students slept longer, spent more time in SB, and less time in MVPA during the COVID-19 measures that included online-schooling. Students spent more time in SB during leisure, as well as during lecture time. Interventions aiming to combat the unfavourable changes in movement behaviours of the youth related to COVID-19 measures are warranted. Teachers and parents/guardians should support and encourage students to sit less and move more, and countries and policies should provide a safe space where youth can be active. Future studies should develop and evaluate the interventions suitable for the epidemic that will decrease SB and, particularly, increase MVPA.

## Figures and Tables

**Table 1:** Participant characteristics (*n* = 51).

Characteristic	*n* (%)
Year of high school	
1st year class	17 (33)
2nd year class	14 (28)
3rd year class	20 (39)
Sex	
Female	26 (51)
Male	25 (49)
zBMI	
Thinness	1 (2)
Normal	44 (86)
Overweight or obese	6 (12)
Smoking status	
Non-smoker	45 (88)
Smoker	6 (12)
Place of residence	
Urban	31 (61)
Rural	20 (39)
Socio-economic status	
High or very high	8 (16)
Middle	41 (80)
Low or very low	2 (4)

Abbreviations: zBMI, body mass index z-score.

**Table 2:** Compositional means of movement behaviours within the onsite-schooling and online-schooling condition for a 4-part composition and for a 6-part composition, mean first pivot coordinates (ilr1) for each corresponding compositional/sub-compositional part, and results of the univariable post hoc tests performed on ilr1.

	Onsite-Schooling Weekdays	Online-Schooling Weekdays	*t* ^a^	MD [95% CI] ^a^	*p* ^a^	*d* ^a^
**4-part composition**						
SL (min/day)	431.9	468.9				
ilr1_SL_	0.824	1.109	6.830	0.285 [0.201, 0.369]	<0.000	0.956
SB (min/day)	730.5	755.0				
ilr1_SB_	1.430	1.659	4.781	0.228 [0.132, 0.324]	<0.000	0.669
LPA (min/day)	252.5	201.5				
ilr1_LPA_	0.204	0.133	−1.75	−0.070 [−0.150, 0.010]	0.086	−0.245
MVPA (min/day)	25.2	14.5				
ilr1_MVPA_	−2.458	−2.901	−4.106	−0.444 [−0.661, −0.227]	<0.000	−0.575
**6-part composition**						
SL (min/day)	436.6	473.2				
**Leisure (3-part sub-composition)**						
SB_leisure_ (min/day)	478.4	491.8				
ilr1_SB leisure_	1.758	1.962	2.826	0.204 [0.059, 0.349]	0.007	0.396
LPA_leisure_ (min/day)	184.3	159.2				
ilr1_LPA leisure_	0.590	0.581	−0.182	−0.009 [−0.107, 0.089]	0.856	−0.026
MVPA_leisure_ (min/day)	16.7	12.4				
ilr1_MVPA leisure_	−2.347	−2.543	−1.829	−0.195 [−0.410, 0.019]	0.073	−0.256
**Lecture (2-part sub-composition)**						
SB_lecture_ (min/day)	252.9	264.0				
ilr1_SB lecture_	0.896	1.344	7.027	0.448 [0.320, 0.576]	<0.000	0.984
PA_lecture_ (min/day)	71.2	39.4				
ilr1_PA lecture_	−0.896	−1.344	−7.027	−0.447 [−0.576, −0.320]	<0.000	−0.984

Abbreviations: SL, sleep; SB, sedentary behaviour; LPA, light physical activity; MVPA, moderate-to-vigorous physical activity; PA, physical activity; MD, mean difference; CI, confidence interval; ilr, isometric log ratio.; ^a^ value from the paired sample *t*-test, where the first pivot coordinate was used to represent each movement behaviour variable.

## Data Availability

The data presented in this study are available on request from the corresponding author.

## References

[1] Ghebreyesus T.A. (2020). WHO Director-General’s Opening Remarks at the Media Briefing on COVID-19. https://www.who.int/director-general/speeches/detail/who-director-general-s-opening-remarks-at-the-media-briefing-on-COVID-19-11-march-2020.

[2] Flaxman S., Mishra S., Gandy A., Unwin H.J.T., Mellan T.A., Coupland H., Whittaker C., Zhu H., Berah T., Eaton J.W. (2020). Estimating the effects of non-pharmaceutical interventions on COVID-19 in Europe. Nature.

[3] Paterson D.C., Ramage K., Moore S.A., Riazi N., Tremblay M.S., Faulkner G. (2021). Exploring the impact of COVID-19 on the movement behaviors of children and youth: A scoping review of evidence after the first year. J. Sport Health Sci..

[4] Chew H.S.J., Lopez V. (2021). Global Impact of COVID-19 on Weight and Weight-Related Behaviors in the Adult Population: A Scoping Review. Int. J. Environ. Res. Public Health.

[5] Cheval B., Sivaramakrishnan H., Maltagliati S., Fessler L., Forestier C., Sarrazin P., Orsholits D., Chalabaev A., Sander D., Ntoumanis N. (2021). Relationships between changes in self-reported physical activity, sedentary behaviour and health during the coronavirus (COVID-19) pandemic in France and Switzerland. J. Sports Sci..

[6] Shad B.J., Thompson J.L., Holwerda A.M., Stocks B., Elhassan Y.S., Philp A., Luc J.C., Wallis G.A. (2019). One Week of Step Reduction Lowers Myofibrillar Protein Synthesis Rates in Young Men. Med. Sci. Sports Exerc..

[7] Wolf S., Seiffer B., Zeibig J.M., Welkerling J., Brokmeier L., Atrott B., Ehring T., Schuch F.B. (2021). Is Physical Activity Associated with Less Depression and Anxiety During the COVID-19 Pandemic? A Rapid Systematic Review. Sports Med..

[8] Kemp B.J., Parrish A.M., Batterham M., Cliff D.P. (2020). Participation in Domains of Physical Activity Among Australian Youth During the Transition From Childhood to Adolescence: A Longitudinal Study. J. Phys. Act. Health.

[9] Booth V.M., Rowlands A.V., Dollman J. (2015). Physical activity temporal trends among children and adolescents. J. Sci. Med. Sport.

[10] Couto J.O., Araújo R.H.O., Silva E.C.M., Soares N.M.M., Santos A.E.d., Silva R.J.d.S. (2020). What is the contribution of each physical activity domain to total physical activity in adolescents?. Braz. J. Kinanthropometry Hum. Perform..

[11] Brazendale K., Beets M.W., Weaver R.G., Pate R.R., Turner-McGrievy G.M., Kaczynski A.T., Chandler J.L., Bohnert A., von Hippel P.T. (2017). Understanding differences between summer vs. school obesogenic behaviors of children: The structured days hypothesis. Int. J. Behav. Nutr. Phys. Act..

[12] Beck J., Chard C.A., Hilzendegen C., Hill J., Stroebele-Benschop N. (2016). In-school versus out-of-school sedentary behavior patterns in U.S. children. BMC Obes..

[13] Stockwell S., Trott M., Tully M., Shin J., Barnett Y., Butler L., McDermott D., Schuch F., Smith L. (2021). Changes in physical activity and sedentary behaviours from before to during the COVID-19 pandemic lockdown: A systematic review. BMJ Open Sport Exerc. Med..

[14] López-Valenciano A., Suárez-Iglesias D., Sanchez-Lastra M.A., Ayán C. (2020). Impact of COVID-19 Pandemic on University Students’ Physical Activity Levels: An Early Systematic Review. Front. Psychol..

[15] Schmidt S.C.E., Anedda B., Burchartz A., Eichsteller A., Kolb S., Nigg C., Niessner C., Oriwol D., Worth A., Woll A. (2020). Physical activity and screen time of children and adolescents before and during the COVID-19 lockdown in Germany: A natural experiment. Sci. Rep..

[16] Sellers C., Dall P., Grant M., Stansfield B. (2016). Validity and reliability of the activPAL3 for measuring posture and stepping in adults and young people. Gait Posture.

[17] World Health Organisation BMI-for-Age (5–19 Years). https://www.who.int/tools/growth-reference-data-for-5to19-years/indicators/bmi-for-age.

[18] Arundell L., Salmon J., Koorts H., Contardo Ayala A.M., Timperio A. (2019). Exploring when and how adolescents sit: Cross-sectional analysis of activPAL-measured patterns of daily sitting time, bouts and breaks. BMC Public Health.

[19] Harrington D.M., Dowd K.P., Tudor-Locke C., Donnelly A.E. (2012). A steps/minute value for moderate intensity physical activity in adolescent females. Pediatr. Exerc. Sci..

[20] PAL Technologies Ltd. PALeventanalysis Tool: An Excel Tool That Allows Analysis of activPAL Events File. https://github.com/PALkitchen/eventanalysis.

[21] Haszard J.J., Meredith-Jones K., Farmer V., Williams S., Galland B., Taylor R. (2020). Non-Wear Time and Presentation of Compositional 24-Hour Time-Use Analyses Influence Conclusions About Sleep and Body Mass Index in Children. J. Meas. Phys. Behav..

[22] (2020). The R Stats Package.

[23] (2020). RStudio: Integrated Development Environment for R.

[24] Gupta N., Mathiassen S.E., Mateu-Figueras G., Heiden M., Hallman D.M., Jorgensen M.B., Holtermann A. (2018). A comparison of standard and compositional data analysis in studies addressing group differences in sedentary behavior and physical activity. Int. J. Behav. Nutr. Phys. Act..

[25] Dumuid D., Stanford T.E., Martin-Fernandez J.A., Pedisic Z., Maher C.A., Lewis L.K., Hron K., Katzmarzyk P.T., Chaput J.P., Fogelholm M. (2017). Compositional data analysis for physical activity, sedentary time and sleep research. Stat. Methods Med. Res..

[26] Dumuid D., Pedišić Ž., Palarea-Albaladejo J., Martín-Fernández J.A., Hron K., Olds T. (2020). Compositional Data Analysis in Time-Use Epidemiology: What, Why, How. Int. J. Environ. Res. Public Health.

[27] Martín-Fernández J.A., Daunis-I-estadella J., Mateu-Figueras G. (2015). On the interpretation of differences between groups for compositional data. SORT.

[28] Pedišić Ž., Dumuid D., Olds T.S. (2017). Integrating sleep, sedentary behaviour, and physical activity research in the emerging field of time-use epidemiology: Definitions, concepts, statistical methods, theoretical framework, and future directions. Kinesiology.

[29] Egozcue J.J., Pawlowsky-Glahn V., Mateu-Figueras G., Barceló-Vidal C. (2003). Isometric Logratio Transformations for Compositional Data Analysis. Math. Geol..

[30] Brusaca L.A., Barbieri D.F., Mathiassen S.E., Holtermann A., Oliveira A.B. (2021). Physical Behaviours in Brazilian Office Workers Working from Home during the COVID-19 Pandemic, Compared to before the Pandemic: A Compositional Data Analysis. Int. J. Environ. Res. Public Health.

[31] Cohen J. (1988). Statistical Power Analysis for the Behavioral Sciences.

[32] Dragun R., Veček N.N., Marendić M., Pribisalić A., Đivić G., Cena H., Polašek O., Kolčić I. (2020). Have Lifestyle Habits and Psychological Well-Being Changed among Adolescents and Medical Students Due to COVID-19 Lockdown in Croatia?. Nutrients.

[33] Giuntella O., Hyde K., Saccardo S., Sadoff S. (2021). Lifestyle and mental health disruptions during COVID-19. Proc. Natl. Acad. Sci. USA.

[34] López-Gil J.F., Tremblay M.S., Brazo-Sayavera J. (2021). Changes in Healthy Behaviors and Meeting 24-h Movement Guidelines in Spanish and Brazilian Preschoolers, Children and Adolescents during the COVID-19 Lockdown. Children.

[35] Sañudo B., Fennell C., Sánchez-Oliver A.J. (2020). Objectively-Assessed Physical Activity, Sedentary Behavior, Smartphone Use, and Sleep Patterns Pre- and during-COVID-19 Quarantine in Young Adults from Spain. Sustainability.

[36] Ács P., Prémusz V., Morvay-Sey K., Pálvölgyi Á., Trpkovici M., Elbert G., Melczer C., Makai A. (2020). Effects of COVID-19 on physical activity behavior among university students: Results of a hungarian online survey. Health Probl. Civiliz..

[37] Barkley J.E., Lepp A., Glickman E., Farnell G., Beiting J., Wiet R., Dowdell B. (2020). The Acute Effects of the COVID-19 Pandemic on Physical Activity and Sedentary Behavior in University Students and Employees. Int. J. Exerc. Sci..

[38] Gallè F., Sabella E.A., Ferracuti S., De Giglio O., Caggiano G., Protano C., Valeriani F., Parisi E.A., Valerio G., Liguori G. (2020). Sedentary Behaviors and Physical Activity of Italian Undergraduate Students during Lockdown at the Time of COVID-19 Pandemic. Int. J. Environ. Res. Public Health.

[39] Zhou J., Xie X., Guo B., Pei R., Pei X., Yang S., Jia P. (2021). Impact of COVID-19 Lockdown on Physical Activity Among the Chinese Youths: The COVID-19 Impact on Lifestyle Change Survey (COINLICS). Front. Public Health.

[40] Gallo L.A., Gallo T.F., Young S.L., Moritz K.M., Akison L.K. (2020). The Impact of Isolation Measures Due to COVID-19 on Energy Intake and Physical Activity Levels in Australian University Students. Nutrients.

[41] Zheng C., Huang W.Y., Sheridan S., Sit C.H., Chen X.K., Wong S.H. (2020). COVID-19 Pandemic Brings a Sedentary Lifestyle in Young Adults: A Cross-Sectional and Longitudinal Study. Int. J. Environ. Res. Public Health.

[42] Maher J.P., Hevel D.J., Reifsteck E.J., Drollette E.S. (2021). Physical activity is positively associated with college students’ positive affect regardless of stressful life events during the COVID-19 pandemic. Psychol. Sport Exerc..

[43] Savage M.J., Hennis P.J., Magistro D., Donaldson J., Healy L.C., James R.M. (2021). Nine Months into the COVID-19 Pandemic: A Longitudinal Study Showing Mental Health and Movement Behaviours Are Impaired in UK Students. Int. J. Environ. Res. Public Health.

[44] López-Bueno R., López-Sánchez G.F., Casajús J.A., Calatayud J., Gil-Salmerón A., Grabovac I., Tully M.A., Smith L. (2020). Health-Related Behaviors Among School-Aged Children and Adolescents During the Spanish COVID-19 Confinement. Front. Pediatr..

[45] Munasinghe S., Sperandei S., Freebairn L., Conroy E., Jani H., Marjanovic S., Page A. (2020). The Impact of Physical Distancing Policies During the COVID-19 Pandemic on Health and Well-Being Among Australian Adolescents. J. Adolesc. Health Off. Publ. Soc. Adolesc. Med..

[46] Hughes A.R., Muggeridge D.J., Gibson A.M., Johnstone A., Kirk A. (2016). Objectively Measured Sedentary Time in Children and Their Parents. AIMS Public Health.

[47] Javad Koohsari M., Nakaya T., Shibata A., Ishii K., Oka K. (2021). Working from Home After the COVID-19 Pandemic: Do Company Employees Sit More and Move Less?. Sustainability.

[48] Jurak G., Sorić M., Ocvirk T., Potočnik Ž.L., Meh K., Đurić S., Sember V., Starc G. (2021). Barriers and Determinants of Active Commuting to School in Slovenia. Sustainability.

[49] Hill A.B. (1965). The Environment And Disease: Association or Causation?. Proc. R. Soc. Med..

